# Supplementary material for: Study on characterization of sandstone pore structure and seepage mechanism based on NMR and CT technology
Source: PLoS One. 2026 Jan 8;21(1):e0337448. doi: 10.1371/journal.pone.0337448 (PMC12782397; doi:10.1371/journal.pone.0337448)
Supplement: Attachment — Submitted filename: Response to Reviewers.docx [file pone.0337448.s003.docx]

Dear Editor-in-Chief:

After carefully and meticulously studying the reviewers' comments, the author has thoroughly re-examined the manuscript and made the following revisions and explanations in accordance with the reviewers' opinions and requirements. Additionally, through further analysis and consideration, the author has also revised the parts that the reviewers did not mention but the author believes should be clarified more explicitly. These revisions are highlighted in red for your verification.

**Academic Editor 1**

**Comment 1.** In your Methods section, please provide additional information regarding the permits you obtained for the work. Please ensure you have included the full name of the authority that approved the field site access and, if no permits were required, a brief statement explaining why.

The author agrees with and accepts the expert's suggestions. We clarify as follows: The specimens used in this study were purchased commercially; no additional permissions are required. The purchase invoice is provided in Figure 1.

Figure. 1 Purchase invoice

**Comment 2.** Thank you for stating the following financial disclosure:
“This work is supported by The National Nature Science Foundation of China (No. 52104222, No. 51909224), the Natural Science Foundation Research Project of Shaanxi Province (2021JLM-48, 2025JCBMS-511, 2019JM-182), and the Special Fund for High-level Talents of Xijing University (XJ24B12, XJ18T04).”

Please state what role the funders took in the study. If the funders had no role, please state: "The funders had no role in study design, data collection and analysis, decision to publish, or preparation of the manuscript." If this statement is not correct you must amend it as needed.
Please include this amended Role of Funder statement in your cover letter; we will change the online submission form on your behalf.

The author agrees with and accepts the expert's suggestions.We hereby clarify that all funders played specific roles in this study. Their contributions are detailed below and have been included in the cover letter:Conceptualization: Kaide Liu, Data curation: Kaide Liu, Songxin Zhao, Formal analysis: Kaide Liu, Songxin Zhao, Funding acquisition: Kaide Liu, Investigation:,Songxin Zhao, Methodology: Kaide Liu, Songxin Zhao, Project administration: Kaide Liu, Resources: Kaide Liu, Software: Kaide Liu, Songxin Zhao, Yu Xia, Qiyu Wang, Xinping Wang, Supervision: Kaide Liu, Wenping Yue, Chaowei Sun, Validation: Songxin Zhao ,Visualization: Kaide Liu, Songxin Zhao ,Writing-original draft: Songxin Zhao ,Writing - review & editing: Kaide Liu.

**Comment 3.** Thank you for updating your data availability statement. You note that your data are available within the Supporting Information files, but no such files have been included with your submission. At this time we ask that you please upload your minimal data set as a Supporting Information file, or to a public repository such as Figshare or Dryad.

Please also ensure that when you upload your file you include separate captions for your supplementary files at the end of your manuscript.
As soon as you confirm the location of the data underlying your findings, we will be able to proceed with the review of your submission.

Thank you for the editor’s comment. The “minimal data set” has now been uploaded as part of the Supporting Information file. A separate heading for the supplementary file has also been added at the end of the manuscript.

**Comment 4.** We note that your manuscript is not formatted using one of PLOS ONE’s accepted file types. Please reattach your manuscript as one of the following file types: .doc, .docx, .rtf, or .tex (accompanied by a .pdf).
If your submission was prepared in LaTex, please submit your manuscript file in PDF format and attach your .tex file as “other.”

Thank you for the editor’s comment. The file has been converted to .docx format.

**Academic Editor 2**

**Comment 1.** Please ensure that your manuscript meets PLOS ONE's style requirements, including those for file naming. The PLOS ONE style templates can be found at https://journals.plos.org/plosone/s/file?id=wjVg/PLOSOne_formatting_sample_main_body.pdf and <https://journals.plos.org/plosone/s/file?id=ba62/PLOSOne_formatting_sample_title_authors_affiliations.pdf.>

The author agrees with and accepts the expert's suggestions, and has made the necessary revisions.

**Comment 2.** In your Methods section, please provide additional information regarding the permits you obtained for the work. Please ensure you have included the full name of the authority that approved the field site access and, if no permits were required, a brief statement explaining why.

Answer in line with Editor 1’ s Comment 1.

**Comment 3.** We note that this submission includes NMR spectroscopy data. We would recommend that you include the following information in your methods section or as Supporting Information files:

1) The make/source of the NMR instrument used in your study, as well as the magnetic field strength. For each individual experiment, please also list: the nucleus being measured; the sample concentration; the solvent in which the sample is dissolved and if solvent signal suppression was used; the reference standard and the temperature.

2) A list of the chemical shifts for all compounds characterised by NMR spectroscopy, specifying, where relevant: the chemical shift (δ), the multiplicity and the coupling constants (in Hz), for the appropriate nuclei used for assignment.

3)The full integrated NMR spectrum, clearly labelled with the compound name and chemical structure.

We also strongly encourage authors to provide primary NMR data files, in particular for new compounds which have not been characterised in the existing literature. Authors should provide the acquisition data, FID files and processing parameters for each experiment, clearly labelled with the compound name and identifier, as well as a structure file for each provided dataset.

See our list of recommended repositories here: https://journals.plos.org/plosone/s/recommended-repositories

The author agrees with and accepts the expert's suggestions. Low-field nuclear magnetic resonance (LF-NMR) was employed in this study to characterize the water distribution and porosity of the sandstone pore structure; no elucidation of organic molecular structures was involved. Consequently, conventional high-resolution NMR parameters—such as chemical shifts, coupling constants, solvents, or reference standards—are not required. All raw data have been provided in the Supporting Information.

Thank you for stating the following financial disclosure:

**Comment 4.** “This work is supported by The National Nature Science Foundation of China (No. 52104222, No. 51909224), the Natural Science Foundation Research Project of Shaanxi Province (2021JLM-48, 2025JCBMS-511, 2019JM-182), and the Special Fund for High-level Talents of Xijing University (XJ24B12, XJ18T04).”

Please state what role the funders took in the study. If the funders had no role, please state: "The funders had no role in study design, data collection and analysis, decision to publish, or preparation of the manuscript." If this statement is not correct you must amend it as needed.

Please include this amended Role of Funder statement in your cover letter; we will change the online submission form on your behalf.

Answer in line with Editor 1’ s Comment 2.

**Comment 5.** We note that your Data Availability Statement is currently as follows:

“All relevant data are within the manuscript and its Supporting Information files.”

Please confirm at this time whether or not your submission contains all raw data required to replicate the results of your study. Authors must share the “minimal data set” for their submission. PLOS defines the minimal data set to consist of the data required to replicate all study findings reported in the article, as well as related metadata and methods (https://journals.plos.org/plosone/s/data-availability#loc-minimal-data-set-definition).

For example, authors should submit the following data:

- The values behind the means, standard deviations and other measures reported;
- The values used to build graphs;
- The points extracted from images for analysis.

Authors do not need to submit their entire data set if only a portion of the data was used in the reported study.

If your submission does not contain these data, please either upload them as Supporting Information files or deposit them to a stable, public repository and provide us with the relevant URLs, DOIs, or accession numbers. For a list of recommended repositories, please see https://journals.plos.org/plosone/s/recommended-repositories.

If there are ethical or legal restrictions on sharing a de-identified data set, please explain them in detail (e.g., data contain potentially sensitive information, data are owned by a third-party organization, etc.) and who has imposed them (e.g., an ethics committee). Please also provide contact information for a data access committee, ethics committee, or other institutional body to which data requests may be sent. If data are owned by a third party, please indicate how others may request data access.

The author agrees with and accepts the expert's suggestions. The minimal data set has been uploaded.

**Comment 6.** We note that Figure 8 in your submission contain copyrighted images. All PLOS content is published under the Creative Commons Attribution License (CC BY 4.0), which means that the manuscript, images, and Supporting Information files will be freely available online, and any third party is permitted to access, download, copy, distribute, and use these materials in any way, even commercially, with proper attribution. For more information, see our copyright guidelines: <http://journals.plos.org/plosone/s/licenses-and-copyright.>

We require you to either (1) present written permission from the copyright holder to publish these figures specifically under the CC BY 4.0 license, or (2) remove the figures from your submission:

1) You may seek permission from the original copyright holder of Figure 8 to publish the content specifically under the CC BY 4.0 license.

We recommend that you contact the original copyright holder with the Content Permission Form (http://journals.plos.org/plosone/s/file?id=7c09/content-permission-form.pdf) and the following text:

“I request permission for the open-access journal PLOS ONE to publish XXX under the Creative Commons Attribution License (CCAL) CC BY 4.0 (http://creativecommons.org/licenses/by/4.0/). Please be aware that this license allows unrestricted use and distribution, even commercially, by third parties. Please reply and provide explicit written permission to publish XXX under a CC BY license and complete the attached form.”

Please upload the completed Content Permission Form or other proof of granted permissions as an ""Other"" file with your submission.

In the figure caption of the copyrighted figure, please include the following text: “Reprinted from [ref] under a CC BY license, with permission from [name of publisher], original copyright [original copyright year].”

2) If you are unable to obtain permission from the original copyright holder to publish these figures under the CC BY 4.0 license or if the copyright holder’s requirements are incompatible with the CC BY 4.0 license, please either i) remove the figure or ii) supply a replacement figure that complies with the CC BY 4.0 license. Please check copyright information on all replacement figures and update the figure caption with source information. If applicable, please specify in the figure caption text when a figure is similar but not identical to the original image and is therefore for illustrative purposes only.

The author agrees with and accepts the expert's suggestions. The figure has been removed.

**Comment 7.** If the reviewer comments include a recommendation to cite specific previously published works, please review and evaluate these publications to determine whether they are relevant and should be cited. There is no requirement to cite these works unless the editor has indicated otherwise.

The author agrees with and accepts the expert's suggestions. The reviewers did not request citation of any specific references.

**Comment 8.** Please review your reference list to ensure that it is complete and correct. If you have cited papers that have been retracted, please include the rationale for doing so in the manuscript text, or remove these references and replace them with relevant current references. Any changes to the reference list should be mentioned in the rebuttal letter that accompanies your revised manuscript. If you need to cite a retracted article, indicate the article’s retracted status in the References list and also include a citation and full reference for the retraction notice.

[Note: HTML markup is below. Please do not edit.]

The author agrees with and accepts the expert's suggestions. All references are complete and accurate.

**Reviewer 1**

**Comment 1.** The figures displaying experimental samples lack a scale bar.

The author agrees with and accepts the expert's suggestions. The specific modifications in the text are as follows:

**2. Experimental methods**

**2.1. NMR experiments**

The detailed NMR experimental steps are shown in Fig 1, specifically as follows: ① Sample origin: Core samples of Luhe Formation sandstone were taken from the west wing of Panel 1 in Gaojiapu Coal Mine, Binchang Mining Area. Among them, coarse and medium sandstones were from the middle section of the Luhe Formation, while fine sandstone was from the lower section, with colors mainly light purplish red and light brownish red. ② Sample preparation: Using a Z3032X8 radial drilling machine and an SPQJ-300 slicing machine, the cores were processed into cylindrical samples with a diameter of 25 mm and a height of 50 mm. The coarse, medium, and fine sandstones were labeled as CSY, ZSY, and XSY, respectively, arranged from left to right as CSY, ZSY, and XSY. ③ Vacuum saturation: The samples were placed in a ZYB-Ⅱ vacuum pressure saturation device, first vacuumed for 8 hours, then water was injected and pressurized to 8 MPa and maintained for more than 24 hours to ensure full saturation of pores. ④ NMR measurement: The samples were placed into a MacroMR12-150H-I low-field nuclear magnetic resonance instrument (Xijing University), and the equipment parameters were set as follows: 25 mm coil, CPMG sequence, O1=12773.43 Hz, P1=8 μs, P2=14.48 μs, SW=250KHz, PRG=2, TW=1500 ms, NS=8, TE=0.06 ms, NECH echo number=18000, to obtain the *T*_2_ spectrum under saturated conditions. ⑤ Centrifugation treatment: The samples were placed in an H3-18K desktop high-speed centrifuge, set at 4000 r/min, and centrifuged for 15 minutes to remove movable water; then, they were tested again to obtain the *T*_2_ spectrum of bound fluid.

**Fig 1. NMR experimental procedure**

**2.2 CT Experiments**

The detailed CT experimental steps are shown in Fig 2 and are as follows: ① Sample origin: identical to those used in the NMR test. ② Sample preparation: cores were first rough-cut with an SPQJ-300 diamond saw, further trimmed with a metallographic cutter, and finally machined into 5 mm × 5 mm × 15 mm rectangular prisms using an LC-200XP automatic high-precision cutter (Nabo, Jiaxing). From left to right, the nine specimens comprised three fine-, three medium-, and three coarse-sandstone prisms; defect-free samples were selected for each lithotype and coded XSY, ZSY, and CSY, respectively. ③ Sample loadting: prisms were loaded into a Zeiss Xradia 510 Versa high-resolution 3D X-ray microscope (Guilin University of Technology) operated with (1) 3-D spatial resolution < 0.7 µm, (2) X-ray tube voltage 30–160 kV, maximum power 10 W, (3) 2k × 2k CCD camera, (4) interchangeable objective lenses (0.4X, 4X) manually selected according to resolution requirements, (5) detector travel range 290 mm, (6) propagation-based phase-contrast imaging, (7) maximum sample width 300 mm, and (8) maximum sample mass (including holder) 15 kg. ④ Test initiated: continuous transverse sections were scanned for each sandstone prism, yielding 1000 two-dimensional sequential images (TIFF format).

**Fig 2. CT experimental procedure**

**(As marked in red in the text, see lines 111 to 155)**

**Comment 2.** Regarding the images of instruments and experimental processes, it is recommended to further integrate them through logical link diagrams or flowcharts.

The revision is identical to Comment 1.

**Comment 3.** This manuscript provides a detailed analysis of the experiment, but what kind of patterns can be obtained from the data obtained? New acquaintance? How to guide problems in production practice? The discussion on these aspects is relatively lacking, it is suggested to further supplement.

The author agrees with and accepts the expert's suggestions. The specific modifications in the text are as follows:

**5.2.3 Significance of micro-pore structure and seepage mechanism investigation**

By analyzing the micro-pore structure and seepage mechanism of different grain-size sandstones in the Luohe Formation of the Gaojiapu coal mine, Huanglong Jurassic coalfield, Ordos Basin, this study clarifies the intrinsic relationship between sandstone pore characteristics and flow capacity, providing key theoretical support and technical guidance for surface pre-grouting projects aimed at blocking and reducing roof water in coal mines. In practice, the rule that “coarse and medium sandstones, with their strong pore connectivity and well-developed throats, constitute the main migration pathways for roof water, whereas fine sandstones contain mostly isolated or poorly connected pores and contribute little to flow” should be used to focus grouting on coarse and medium sandstones while adopting simplified treatment for fine sandstones to avoid cost waste. For material and technique selection, high-fill composite slurries should be chosen for the macro-pore–fracture system of coarse and medium sandstones, and low-viscosity, high-penetration slurries for the micro-pores of fine sandstones; a staged pressure regime of “low-pressure diffusion plus medium-pressure consolidation” can balance spread radius and filling density. Meanwhile, borehole layout and construction monitoring should be optimized according to the different seepage responses: in coarse and medium sandstone zones, flow-rate and pressure sensors should be prioritized for early warning of water-inrush risk, whereas in fine sandstone zones, water-level tracking should be emphasized to judge plugging effectiveness. Integrating these findings into engineering design enables a scientifically based grouting scheme that ensures safe coal extraction while reducing both project cost and management blindness.

**(As marked in red in the text, see lines 672 to 695)**

**Comment 4.** It is suggested that the authors further optimize the language and supplement the references from the past three years.

The author agrees with and accepts the expert's suggestions. The specific modifications in the text are as follows:

Roof–water inrush has long been a critical geological barrier to the safe and high–efficiency extraction of coal resources [1,2]. The Huanglong Jurassic coalfield in the Ordos Basin, one of China’s major energy bases, is threatened by the huge Luohe Formation sandstone aquifer that directly overlies the workable seams [3,4]. To tackle this problem, previous studies have systematically documented lithofacies characteristics, rock mechanical properties, and the height of the “two–zone” (caving + fractured) development [4-11]. It has been demonstrated that the primary fissures embedded in the water–bearing sandstone provide the essential skeleton for the initiation and propagation of water-conducting fracture networks [11], while the micro-porous architecture of the sandstone itself offers the necessary storage space and flow pathways for confined groundwater [6]. Therefore, systematically and comprehensively understanding the characteristics of the microscopic pore structure and the seepage mechanism of the thick water-bearing Luhe Formation sandstone is also a key part of accurately formulating the prevention and control plan for the roof sandstone water hazard, and it is of great significance for effectively preventing and controlling the roof sandstone water hazard.

Extensive studies worldwide have focused on the micro-pore architecture and flow mechanisms of sandstones. Regarding pore-structure characterization, Wang et al. [12] combined NMR with fractal theory and demonstrated that freeze–thaw cycles enlarge macropores and throats, leading to a linear increase in permeability and a reduction in fractal dimension. This identifies macropores as the most freeze-sensitive feature. Mu et al. [13] introduced a trimodal NMR-porosity index and a throat-radius index to quantify the complex pore network of tight Chang-8 and Chang-9 sandstones in the Zhijing–Ansai area of the Ordos Basin, markedly improving the accuracy of log interpretation. Mondal et al. [14] employed NMR to characterize the pore structure of complex Eocene carbonates from an offshore oilfield in India. By correlating NMR *T*₂ distributions with capillary-pressure data, they derived a conversion coefficient, performed radius-dependent porosity partitioning, and conducted a two-stage fractal analysis, revealing a clear relationship between fractal dimension and petrophysical parameters. Zhang et al. [15] used mercury-injection capillary pressure (MIP) and NMR to analyze the pore structure of coal-measure sedimentary rocks (shale, mudstone, and sandstone) and determined pore-size distributions and fractal dimensions. Zhao et al. [16] utilized X-ray computed tomography (CT) to realize three-dimensional visualization and refined quantitative characterization of rock micro-structures. Zheng et al. [17] combined micro-CT digital-core technology with Avizo-based algorithms to construct digital-core models, enabling three-dimensional visualization and quantitative analysis of the micro-pore structure of tight sandstone samples from the Stone-Box Formation in the northern J area of the Ordos Basin, thereby providing technical support for quantitative evaluation of reservoir micro-parameters and for visualization and quantitative characterization of sample micro-structures.

In terms of flow mechanisms, Wang et al. [18] employed CT scanning to investigate the pore structure and mechanical properties of porous rock in mining strata, revealing spatial consistency between the pore network model (PNM) and crack propagation paths; by integrating a network model with pore topology, they enabled pore-scale simulation and prediction of permeability. Wang et al. [19] acquired sandstone pore-structure data via CT and performed multiscale flow simulations with network models to clarify how connectivity, throat-size distribution, and grain arrangement control fluid movement. Zhao et al. [20] combined CT with pore-network modeling to quantify the impact of topological parameters such as connectivity and branch number on permeability in sandstone reservoirs and proposed an improved pore-network simulation method. Liu et al. [21] extracted pore structures from CT images, predicted flow capacity with network models, examined the influence of connectivity and branch number on permeability, and presented an upgraded network approach. Zhang et al. [22] constructed pore-network models from CT data for high-porosity sandstones, analyzed fluid flow, studied the effects of connectivity and throat-size distribution on permeability, and developed an improved network algorithm. Liu et al. [23] built equivalent pore-network models from micro-CT images using image-processing algorithms, realized pore-scale flow simulations, and computed absolute permeability, offering new insights into sandstone flow behavior. Liu et al. [24] reconstructed micro-pore geometries directly from image files with COMSOL and conducted pore-scale flow simulations, validating the feasibility of image-based geometric reconstruction and providing a novel method for investigating micro-flow mechanisms in low-permeability reservoirs.

Although abundant studies have addressed sandstone pore structure and flow behavior, the two aspects are usually treated separately, and comparative analyses of different sandstone types within a single stratigraphic unit remain scarce and fragmentary. To fill this gap, we present an integrated investigation of coarse, medium, and fine sandstones widely occurring in the Luohe Formation of the Gaojiapu coal mine, Huanglong Jurassic coalfield, Ordos Basin. NMR and X-CT experiments were combined with Avizo, Netfabb, and COMSOL workflows to characterize pore architecture and pore-scale flow, aiming to provide a theoretical basis for roof-water-inrush prevention. The Luohe sandstone aquifer is exceptionally thick (average 400.35 m) and highly water-rich (maximum specific capacity 2.288 L s⁻¹ m⁻¹). The parting between the aquifer and the coal seam is only ~84 m, giving rise to a measured water pressure of 7.4 MPa. The upper-middle and lower-middle sub-sections are the main water-bearing intervals, yet they are neither drainable nor easily groutable because of low fracture intensity, limited grout take, and a diffusion radius of only a few meters, posing a serious challenge to water-pressure reduction by grouting.

**(As marked in red in the text, see lines 31 to 110)**

**Reviewer 2**

**Comment 1.** This study employs NMR and CT techniques to quantitatively compare the porosity, pore-size distribution, pore type, connectivity, and permeability of different sandstones. Digital cores reconstructed from CT slices are then used to perform pore-scale flow simulations in Comsol, providing a theoretical basis for preventing roof-water hazards in coal seams.

Thank you for your positive feedback.

**Comment 2.** Title ambiguity: change “Study on Characterization of Sandstone Pore Structure and Permeability Simulation Based on NMR and CT Technology” to “Study on Characterization of Sandstone Pore Structure and Seepage Mechanism Based on NMR and CT Technology”.

The author agrees with and accepts the expert's suggestions. The specific modifications in the text are as follows:

Study on Characterization of Sandstone Pore Structure and Seepage Mechanism Based on NMR and CT Technology

**(As marked in red in the text, see lines 4 to 6)**

**Comment 3.** The abstract is verbose and imprecise; condense it to highlight purpose, methods, conclusions, and engineering significance while tightening the logic.

The author agrees with and accepts the expert's suggestions. The specific modifications in the text are as follows:

Coal-seam roof water disasters severely constrain safe and high-efficiency coal production. Integrating NMR, X-CT, Avizo, Netfabb, and Comsol, we systematically characterized pore architecture and flow behavior for the coarse, medium, and fine sandstones of the Luohe Formation in the Gaojiapu coal mine, Huanglong Jurassic coalfield, Ordos Basin. Results show that the NMR cumulative porosity of coarse, medium, and fine sandstones is 14.36 %, 17.82 %, and 16.09 %, respectively; NMR permeability is 38.77 mD, 1.46 mD, and 0.87 mD; the proportions of macropores and micro-fractures are 48 % and 27 %, 34 % and 12 %, and 46 % and 8 %; fully movable porosity accounts for 68 %, 42 %, and 29 %; NMR-connected pore fractal dimensions are 2.847, 2.943, and 2.955; CT total porosity is 16.43 %, 15.51 %, and 14.68 %; CT connected porosity is 16.19 %, 15.21 %, and 14.36 %; connectivity is 98.54 %, 98.07 %, and 97.82 %; mean pore diameter is 17.50 μm, 17.16 μm, and 17.03 μm; mean throat radius is 32.63 μm, 29.11 μm, and 28.75 μm; absolute permeability is 10.88 D, 10.56 D, and 8.40 D; tortuosity is 1.63, 1.65, and 1.69. Flow-field simulations indicate that velocities below 0.08 m/s dominate, covering 80.14 % in coarse sandstone and 97.54 % in fine sandstone, providing a theoretical basis for roof water-hazard prevention.

**(As marked in red in the text, see lines 13 to 29)**

**Comment 4.** Lines 58~60 of the Introduction (“Existing grouting has sealed most pores and fractures; however, tiny pores still leak continuously, so sandstone-roof water hazards remain serious”) require literature support.

The revision follows Expert 1’s Comment 4 exactly.

**Comment 5.** The Introduction should summarize the limitations of previous work on pore structure and seepage mechanism, with citations, to strengthen the motivation for this study.

The revision follows Expert 1’s Comment 4 exactly.

**Comment 6.** Add a brief description of the geological setting of the tested samples at the end of the Introduction.

The revision follows Expert 1’s Comment 4 exactly.

**Comment 7.** Sections 3 and 4.1~4.2 analyze porosity, permeability, fractal dimension, tortuosity, etc., but the theoretical link between pore structure and seepage mechanism is unclear; insert a new discussion section before the flow simulations.

The author agrees with and accepts the expert's suggestions. The specific modifications in the text are as follows:

5. Seepage mechanism and numerical simulation

Pore-scale flow physics governs fluid transport in sandstone reservoirs. Here we focus on how the reconstructed pore architecture controls single-phase flow and simulate it with a creeping-flow model (Navier–Stokes equations).

**5.1 Fundamental control of pore structure on seepage mechanisms**

The pore structure of sandstone (pore-size distribution, connectivity, topological morphology, and fractal characteristics) directly dominates the differences in seepage mechanisms by altering flow paths, resistance, and fluid storage state. Combined NMR and X-CT data reveal the following specific controls for the three lithotypes: ① Pore size determines fluid mobility patterns. NMR measurements show that coarse sandstone macropores and micro-fractures account for 48 % and fully movable porosity for 68 %. The large pore space provides ample room for fluid migration, allowing water molecules to form "preferential channel flow" along continuous conduits, yielding a permeability of 38.77 mD. Fine sandstone exhibits 19 % micropores and 27 % small pores with 64 % immovable fraction; fluids are mostly adsorbed on pore surfaces or trapped in isolated spaces, migrating only through "slow diffusion under viscous control", giving a permeability of merely 0.87 mD. Medium sandstone contains 34 % mesopores and 28 % micropores, displaying a mixed "preferential channel flow plus local diffusion" regime with 1.46 mD permeability, confirming that larger pores correlate with higher movable-fluid fraction and greater flow efficiency. ② Pore connectivity governs seepage pathways. 3-D CT reconstructions indicate coarse sandstone has 16.19 % connected porosity and 98.54 % connectivity, forming a "densely interconnected mesh" with a mean throat radius of 32.63 µm and tortuosity of 1.63, offering straight flow paths. Fine sandstone shows 14.36 % connected porosity and 97.82 % connectivity, with pores mostly "isolated dots or short connections", mean throat radius 28.75 µm, and tortuosity 1.69, forcing fluid to detour and markedly increasing resistance. Average coordination numbers further verify this trend, being 8.05, 7.83, and 7.65 for coarse, medium, and fine sandstones, respectively; higher coordination yields more effective flow channels and stronger seepage capacity. ③ Fractal characteristics influence flow heterogeneity. NMR-derived fractal dimensions of connected pores are 2.955 for fine sandstone, 2.943 for medium sandstone, and 2.847 for coarse sandstone. The higher fractal dimension reflects stronger structural heterogeneity, meaning greater contrasts in pore size and more uneven spatial distribution, causing more frequent "sudden changes in flow resistance" during fluid migration and consequently the poorest seepage performance.

**(As marked in red in the text, see lines 538 to 574)**

**Comment 8.** Formatting errors (lines 359, 377~387, 423, 429, 486), such as misaligned equations, must be corrected throughout.

The author agrees with and accepts the expert's suggestions. The specific modifications in the text are as follows:

$\frac{\varphi_{NMR,T_{2cutoff}}-\varphi_{wi,T_{2cutoff}}}{\varphi_{\mathrm{NMR}}-\varphi_{\mathrm{wi}}}>0.01$	（2）

$K_{Coates-cutoff}=\left( \frac{\varphi_{\mathrm{NMR}}}{C_{1}} \right)^{4}\left( \frac{FFI}{BVI} \right)^{2}$	（3）

$K_{Coates-cutoff-REV}=\left( \frac{\varphi_{\mathrm{NMR}}}{C_{2}} \right)^{m}\left( \frac{FFI}{BVI} \right)^{n}$	（4）

$K_{\mathrm{SDR}}=C_{3}\left( \frac{\varphi_{\mathrm{NMR}}}{100} \right)^{2}T_{2g}^{2}$	（5）

$K_{SDR-REV}=C_{4}\left( \frac{\varphi_{\mathrm{NMR}}}{100} \right)^{m}T_{2g}^{n}$	（6）

$T_{2}=\frac{{(c_{1}r)}^{n}}{\rho_{2}F_{s}}$	（8）

$r=CT_{2}^{1/n}$	（9）

$S_{v}=\left( \frac{T_{2max}}{T_{2}} \right)^{D-3}$	（10）

**(As marked in red in the text, see lines 262, 281 ~ 284, 320, 326, 386)**
