# Supplementary material for: Study on characterization of sandstone pore structure and seepage mechanism based on NMR and CT technology
Source: PLoS One. 2026 Jan 8;21(1):e0337448. doi: 10.1371/journal.pone.0337448 (PMC12782397; doi:10.1371/journal.pone.0337448)
Supplement: Attachment — Submitted filename: Response_to_Reviewers_auresp_2.docx [file pone.0337448.s004.docx]

Dear Editor-in-Chief:

After carefully and meticulously studying the reviewers' comments, the author has thoroughly re-examined the manuscript and made the following revisions and explanations in accordance with the reviewers' opinions and requirements. Additionally, through further analysis and consideration, the author has also revised the parts that the reviewers did not mention but the author believes should be clarified more explicitly. These revisions are highlighted in red for your verification.

**Reviewer 1**

**Comment 1.** There is one point raised by Reviewer 1 that was not adequately addressed in the authors’ response. Please review it again:“This manuscript provides a detailed analysis of the experiment, but what kind of patterns can be obtained from the data obtained? New acquaintance? How to guide problems in production practice? The discussion on these aspects is relatively lacking; it is suggested to further supplement.”

The author agrees with and accepts the expert's suggestions. The specific modifications in the text are as follows:

**5.2.3 Significance of micro-pore structure and seepage mechanism investigation**

By analyzing the micro-pore structure and seepage mechanism of different grain-size sandstones in the Luohe Formation of the Gaojiapu coal mine, Huanglong Jurassic coalfield, Ordos Basin, this study clarifies the intrinsic relationship between sandstone pore characteristics and flow capacity, providing key theoretical support and technical guidance for surface pre-grouting projects aimed at blocking and reducing roof water in coal mines. In practice, the rule that “coarse and medium sandstones, with their strong pore connectivity and well-developed throats, constitute the main migration pathways for roof water, whereas fine sandstones contain mostly isolated or poorly connected pores and contribute little to flow” should be used to focus grouting on coarse and medium sandstones while adopting simplified treatment for fine sandstones to avoid cost waste. For material and technique selection, high-fill composite slurries should be chosen for the macro-pore–fracture system of coarse and medium sandstones, and low-viscosity, high-penetration slurries for the micro-pores of fine sandstones; a staged pressure regime of “low-pressure diffusion plus medium-pressure consolidation” can balance spread radius and filling density. Meanwhile, borehole layout and construction monitoring should be optimized according to the different seepage responses: in coarse and medium sandstone zones, flow-rate and pressure sensors should be prioritized for early warning of water-inrush risk, whereas in fine sandstone zones, water-level tracking should be emphasized to judge plugging effectiveness. Integrating these findings into engineering design enables a scientifically based grouting scheme that ensures safe coal extraction while reducing both project cost and management blindness.

**(As marked in red in the text, see lines 672 to 695)**

**Comment 2.** The figures in the paper still do not fully meet standard formatting requirements. Please revise each one accordingly.

The author agrees with and accepts the expert's suggestions. All figures and tables have been reformatted to comply with the journal’s specifications.

**Comment 3.** The references should be standardized. In particular, it is essential to investigate and cite relevant papers published in the last three years.

The author agrees with and accepts the expert's suggestions. The references have been standardized as requested.
